# The complete mitochondrial genome of medicinal fungus *Taiwanofungus camphoratus* reveals gene rearrangements and intron dynamics of *Polyporales*

**DOI:** 10.1038/s41598-020-73461-x

**Published:** 2020-10-05

**Authors:** Xu Wang, Lihua Jia, Mingdao Wang, Hao Yang, Mingyue Chen, Xiao Li, Hanyu Liu, Qiang Li, Na Liu

**Affiliations:** 1 College of Life Sciences, Henan Agricultural University, Zhengzhou, 450002 Henan China; 2 School of Food and Biological Engineering, Chengdu University, Chengdu, 610106 Sichuan China; 3 Present Address: College of Life Sciences, Henan Agricultural University, Zhengzhou, 450002 Henan China

**Keywords:** Fungi, Genome, Genomics

## Abstract

*Taiwanofungus camphoratus* is a highly valued medicinal mushroom that is endemic to Taiwan, China. In the present study, the mitogenome of *T. camphoratus* was assembled and compared with other published *Polyporales* mitogenomes. The *T. camphoratus* mitogenome was composed of circular DNA molecules, with a total size of 114,922 bp. Genome collinearity analysis revealed large-scale gene rearrangements between the mitogenomes of *Polyporales*, and *T. camphoratus* contained a unique gene order. The number and classes of introns were highly variable in 12 *Polyporales* species we examined, which proved that numerous intron loss or gain events occurred in the evolution of *Polyporales*. The Ka/Ks values for most core protein coding genes in *Polyporales* species were less than 1, indicating that these genes were subject to purifying selection. However, the *rps3* gene was found under positive or relaxed selection between some *Polyporales* species. Phylogenetic analysis based on the combined mitochondrial gene set obtained a well-supported topology, and *T. camphoratus* was identified as a sister species to *Laetiporus sulphureus*. This study served as the first report on the mitogenome in the *Taiwanofungus* genus, which will provide a basis for understanding the phylogeny and evolution of this important fungus.

## Introduction

*Taiwanofungus camphoratus*, belonging to *Polyporales*, *Basidiomycota*, is an endemic mushroom of Taiwan, China, which is restricted to the endemic aromatic tree *Cinnamomum kanehirai*^1^. In the last few decades, *T. camphoratus* was included in folk medicine in Asia and has shown remarkable effectiveness in the treatment of inflammatory disorders, cancer, hypertension and hepatitis^2–6^. The total market value of *T. camphoratus* products has grown rapidly, with market value reported to exceed $100 million annually in 2014^7^. Many active secondary metabolites were isolated from the mycelium or fruiting bodies of *T. camphoratus*, such as triterpenoids, lipids, and benzenoids, showing excellent anti-inflammatory, hepatoprotective, antioxidant, radioprotective, and chemoprevention activities^8–11^. *T. camphoratus* shares many common characters with *Antrodia* and *Antrodiella*, which caused its taxonomic confusion in the past^1^. Phylogenetic analysis based on LSU rDNA sequence indicated that *T. camphoratus* should be proposed as a new genus of lignicolous polypore^1^. The genome of *T. camphoratus* has been published to reveal the sexual development and metabolic synthesis of *T. camphoratus*. However, the characterization of the mitogenome of *Taiwanofungus camphoratus* has not been conducted to date.

Mitochondria generate most of the cell's supply of adenosine triphosphate (ATP) in eukaryotes^12^. Most eukaryotes have their own mitochondrial genomes (mitogenomes), which are believed to be derived through endosymbiosis from the ancestral alpha-proteobacterium^13, 14^. As the “second genome” of eukaryotes, mitogenomes have many characteristics different from nuclear genomes. For example, each eukaryotic cell contains a large number of mitochondrial organelles, and each mitochondrial organelle has multiple mitochondrial genomes^15^. In addition, its rapid evolutionary rate, and multiple available molecular markers have made the mitochondrial genome a powerful tool for studying phylogeny and population genetics^16, 17^. With the rapid development of next-generation sequencing technology, more and more mitochondrial genomes have been obtained^18–22^. So far, more than 10,000 complete mitochondrial genomes have been released in the NCBI reference database, including animal, plant and fungal mitogenomes. These available mitogenomes promote the phylogeny and taxonomy of eukaryotes^23–25^. However, compared with its animal counterparts, the mitochondrial genome of fungi has been less studied^26^. As an important group of eukaryotes, fungus is widely distributed in the world. It is estimated that more than 2.2 million of fungal species exist in nature^27^, which play an important role in maintaining the carbon and nitrogen cycle in nature, maintaining the healthy development of forest ecosystems, and providing food sources for human beings^7, 28, 29^. However, fewer than 800 fungal mitogenomes are available, with available mitogenomes of Basidiomycetes are even less (< 130). The few available mitogenomes limit our overall understanding of evolution, phylogeny, and taxonomy of the fungal lineages, because phylogenies based on mitochondrial genes and nuclear genes are not always consistent^30^.

Up to now, only 11 complete mitogenomes of *Polyporales* species have been published, including seven species in *Ganoderma* genus^31–34^, one in *Trametes*^35^, one in *Phlebia*^36^, one in *Laetiporus*^37^, and one in *Fomitopsis*^38^. The sizes of these mitogenomes varied greatly, ranging from 60,635 to 156,348 bp. This is thought to be due to variations of intron numbers. Introns in the mitogenome of fungi can be divided into two groups, namely the group I and the group II. Group II intron excision occurs in the absence of GTP and involves the formation of a lariat, which were different from group I intron. These introns were found distributed in the *cox*, *cob*, *nad* and rRNA genes of the fungal mitogenomes^39–42^, which could modify the organization and size of mitogenome^39, 43, 44^. In addition, a variety of homing endonuclease genes have been found in fungal introns, such as the LAGLIDADG endonuclease and GIY endonucleases^45, 46^, the products of which have homing endonuclease activities. However, the origin, evolution, and dynamics of the mitochondrial introns in the order *Polyporales* are still unknown.

The mitogenomes of fungi are significantly different from that of other eukaryotes. The genome size, gene arrangement, repetitive sequences, introns, intergenic regions, and open reading frames (ORFs) of fungal mitogenomes varied greatly between species and even between intraspecific species^18, 47, 48^. Despite the large variations, most Basidiomycete mitogenomes were found containing 14 core protein coding genes (*atp6*, *atp8*, *atp9*, *cox1*, *cox2*, *cox3*, *cob*, *nad1*, *nad2*, *nad3*, *nad4*, *nad4L*, *nad5*, and *nad6*) for energy metabolism, one *rps3* gene for transcriptional regulation^49^, 22–36 tRNA genes, and two rRNA genes^50, 51^. Repeated sequences in the mitogenome, gene arrangement, and structures of tRNAs can provide important information to reveal the evolutionary and phylogenetic relationships of fungi^39, 52, 53^.

In the present study, the mitogenome of *T. camphoratus* were assembled and annotated. The aims of this study are (1) to reveal variations and conservation of gene content, genome organization, and gene order between *T. camphoratus* mitogenome and other *Polyporales* species; (2) to provide insights into the evolution and dynamic changes of introns in *Polyporales* mitogenomes; (3) to reveal the phylogenetic status of *T. camphoratus* among various *Agaricomycetes* species based on the combined mitochondrial gene set. The mitogenome of *T. camphoratus* will allow further study of the taxonomy, phylogenetics, and evolutionary studies of this important medical fungus and other closely related species.

## Results

### Genome features and protein coding genes

**Figure 1:**
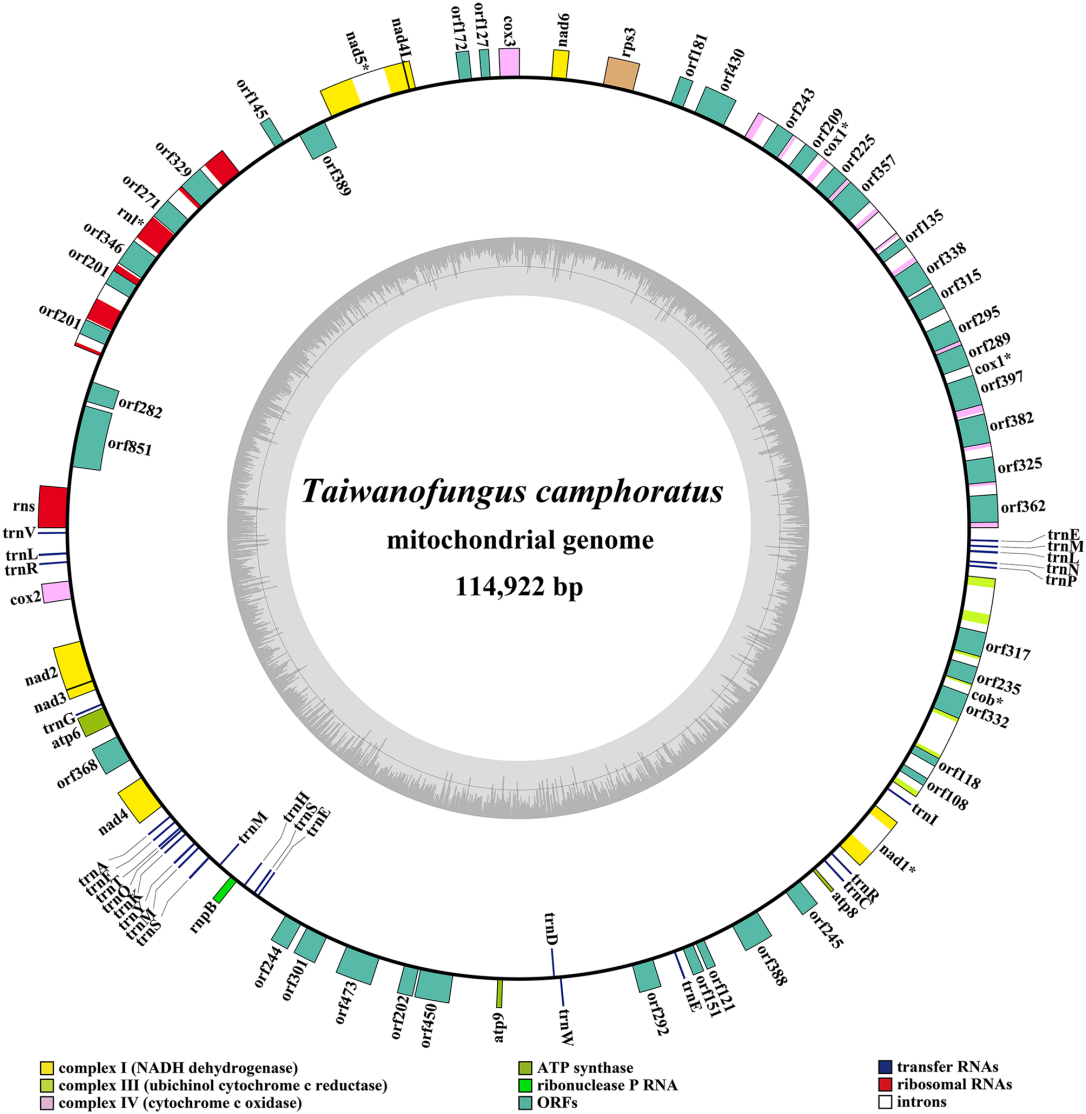
Circular map of the mitochondrial genome of *Taiwanofungus camphoratus*. Genes are represented by different colored blocks. Colored blocks outside each ring indicate that the genes are on the direct strand, while colored blocks within the ring indicates that the genes are located on the reverse strand.

The complete mitogenome of *T. camphoratus* was composed of circular DNA molecules, with the total size of 114,922 bp (Fig. 1). GC content of the *T. camphoratus* mitogenome was 26.01%. The AT skew was negative in the mitogenome of *T. camphoratus*, where the GC skew was positive (Table S1). Fifteen core protein coding genes were detected in the *T. camphoratus* mitogenome, including *atp6*, *atp8*, *atp9*, *cob*, *cox1*, *cox2*, *cox3*, *nad1*, *nad2*, *nad3*, *nad4*, *nad4L*, *nad5*, and *nad6* for energy metabolism, and one *rps3* gene for transcriptional regulation (Table S2). In addition, the *T. camphpratus* mitogenome contained 19 non-intronic ORFs, which included an RNA polymerase gene, two DNA polymerase genes and 16 PCGs with unknown function. Twenty-four introns were detected in the mitogenome of *T. camphoratus*, which were distributed in the host gene of *cox1*, *cob*, *nad1*, and *rnl*. All of these introns belong to the group I. Twenty-three intronic ORFs were found located in these introns, which encoded LAGLIDADG and GIY-YIG homing endonucleases.

### RNA genes in the *T. camphoratus* mitogenome

**Figure 2:**
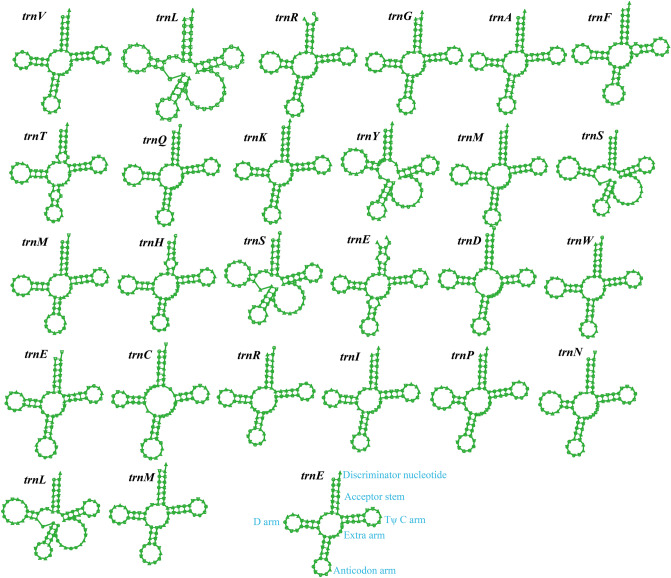
Putative secondary structures of the 27 tRNA genes identified in the mitochondrial genome of *Taiwanofungus camphoratus*. All genes are shown in order of occurrence in the mitochondrial genome of *Taiwanofungus camphoratus*, starting from *trnV.* tRNA structures were determined using MITOS.

Two rRNA genes were detected in the *T. camphoratus* mitogenome, namely the large subunit ribosomal RNA (*rnl*), and the small subunit ribosomal RNA (*rns*) (Table S2). The mitogenome of *T. camphoratus* contained 27 tRNA genes, which were folded into classical cloverleaf structures (Fig. 2). The mitogenome of *T. camphoratus* contained 2 tRNAs with different anticodons coding for serine, arginine, leucine and 3 tRNAs with different anticodons coding for glutamate. In addition, the *T. camphoratus* mitogenome contained 3 tRNAs with the same anticodon that coded for methionine. The length of individual tRNAs ranged from 71 to 86 bp, which was mainly due to the variation of extra arm. A ribonuclease P RNA (*rnpB*) gene was found in the *T. camphoratus* mitogenome, with the length of 329 bp.

### Overlapping nucleotides and mitogenome composition

**Figure 3:**
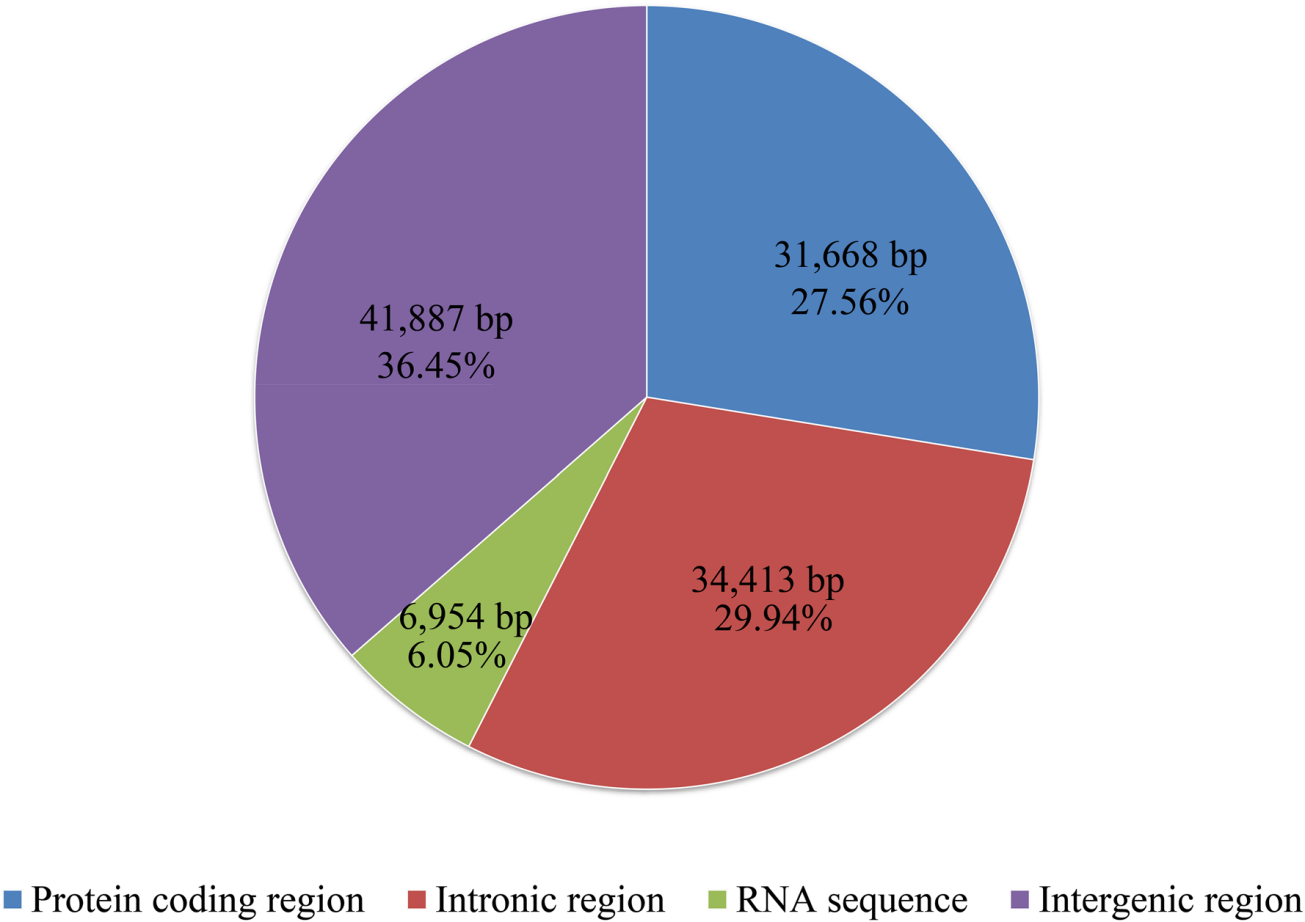
The protein-coding, intronic, intergenic, and RNA gene region proportions of the entire mitochondrial genome of *Taiwanofungus camphoratus*.

Only one overlapping nucleotide was detected in the mitogenome of *T. camphoratus*, which was located across the neighboring genes *nad4L* and *nad5* (1 bp) (Table S2). We detected 41,887 bp intergenic sequences in the mitogenomes of *T. camphoratus*. The length of these intergenic sequences ranged from 0 to 2903 bp, and the longest intergenic sequence was located between the *trnW* and orf292 gene. Intergenic regions accounted for the largest proportion of the *T. camphoratus* mitogenome, reaching 36.45% (Fig. 3), which showed that the mitogenome of *T. camphoratus* had a relatively relaxed structure. The intronic region occupied the second largest proportion of the *T. camphoratus* mitogenome, accounting for 29.94%. The protein coding region accounted for 27.56% of the *T. camphoratus* mitogenome. The RNA region (rRNA + tRNA + *rnpB*) accounted for 6.05% of the entire mitogenome.

### Codon usage analysis

ATG was the most commonly used start codon for the 15 core PCGs in the 12 *Polyporales* species we examined (Table S3). Eleven of the 15 core PCGs used ATG as start codons in all the 12 *Polyporales* mitogenomes we detected. GTG was the most commonly used start codon in *cob* gene of the 12 *Polyporales* mitogenomes. In addition, ATA and TTG were also used as start codons in *cox2*, *nad1* and *nad6* gene of some *Polyporales* species. TAA was most commonly used as stop codons of core PCGs in 12 *Polyporales* species, followed by TTG and AGT. We found that all core PCGs in *T. camphoratus* used ATG as start codons and TAA as stop codons.

**Figure 4:**
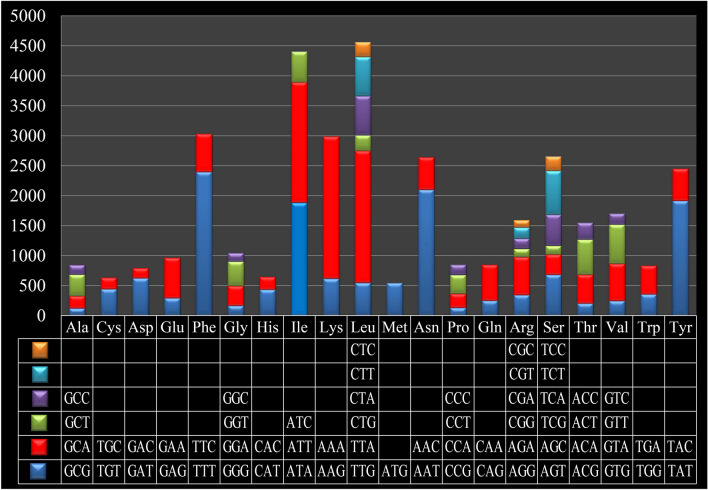
Codon usage in the mitochondrial genome of *Taiwanofungus camphoratus*. Count of codon usage is plotted on the y-axis.

Codon usage analysis indicated that the most frequently used codons in the *T. camphoratus* mitogenome were TTT (for phenylalanine; Phe), AAA (for lycine; Lys), TTA (for leucine; Leu), AAT (for asparagine; Asn), ATT (for isoleucine; Ile), and TAT (for tyrosine; Tyr) (Fig. 4 and Table S4). The frequent use of A and T in codon contributed to the high AT content in the *T. camphoratus* mitogenome (average: 73.99%).

### Repetitive sequences in the mitogenome

Comparing the whole mitogenome of *T. camphoratus* with itself via BLASTn search, we identified 45 repeat sequences in the mitogenome of *T. camphoratus* (Table S5). The length of these repeat sequences ranged from 28 to 902 bp, with pair-wise nucleotide similarities ranging from 74.72 to 100%. The largest repeats were observed in the third introns and fourth introns of the *rnl* gene. The second largest repeats were found located in the intergenic region between orf145 and *rnl*, as well as in intergenic region between orf388 and orf245, with each repeating sequence 840 bp long. Repeat sequence accounted for 9.44% of the *T. camphoratus* mitogenome.

Using the Tandem Repeats Finder, we identified 55 tandem repeats in the *T. camphoratus* mitogenome (Table S6). The longest tandem repeat sequence was observed in the intergenic region between *nad4* and *trnA*, comprising 79 bp. Most of the tandem repeat sequences were duplicated once or twice in the *T. camphoratus* mitogenome, with the highest replication number 15. Tandem repeat sequences accounted for 2.04% of the *T. camphoratus* mitogenome. REPuter identified 27 forward, 7 palindromic, and 16 reverse repeats in the mitogenome of *T. camphoratus*, accounting for 6.33% of the entire mitogenome (Table S7).

### Comparative genome analysis and gene rearrangement

The mitogenome of *T. camphoratus* was the fourth largest among the published mitogenomes in *Polyporales*, which was smaller than *Phlebia radiata*^36^, *G. calidophilum*, and *G. applanatum*, and larger than *Trametes cingulata*^35^, *Fomitopsis palustris*^38^, *Laetiporus sulphureus*^37^ and other *Ganoderma* spp. species (Table S1). The GC content of the *T. camphoratus* mitogenome was in the middle among all *Polyporales* mitogenomes. Of the 12 *Polyporales* we tested, only *T. camphoratus* and *L. sulphureus* had negative AT skews, while the others were positive. The GC skew of the *T. camphoratus* mitogenome was positive, just like most of the *Polyporales*. Thirty-four non-intronic ORFs and 24 introns were found in the *T. camphoratus* mitogenome, which were smaller than the 79 non-intronic ORFs and 36 introns in the *P. radiata* mitogenome that made *P. radiata* the largest mitochondrial genome in the *Polyporales*. All 12 *Polyporales* mitogenomes detected contain 2 rRNA genes. The number of tRNA genes in the mitogenomes of *Polyporales* ranged from 25 to 29.

**Figure 5:**
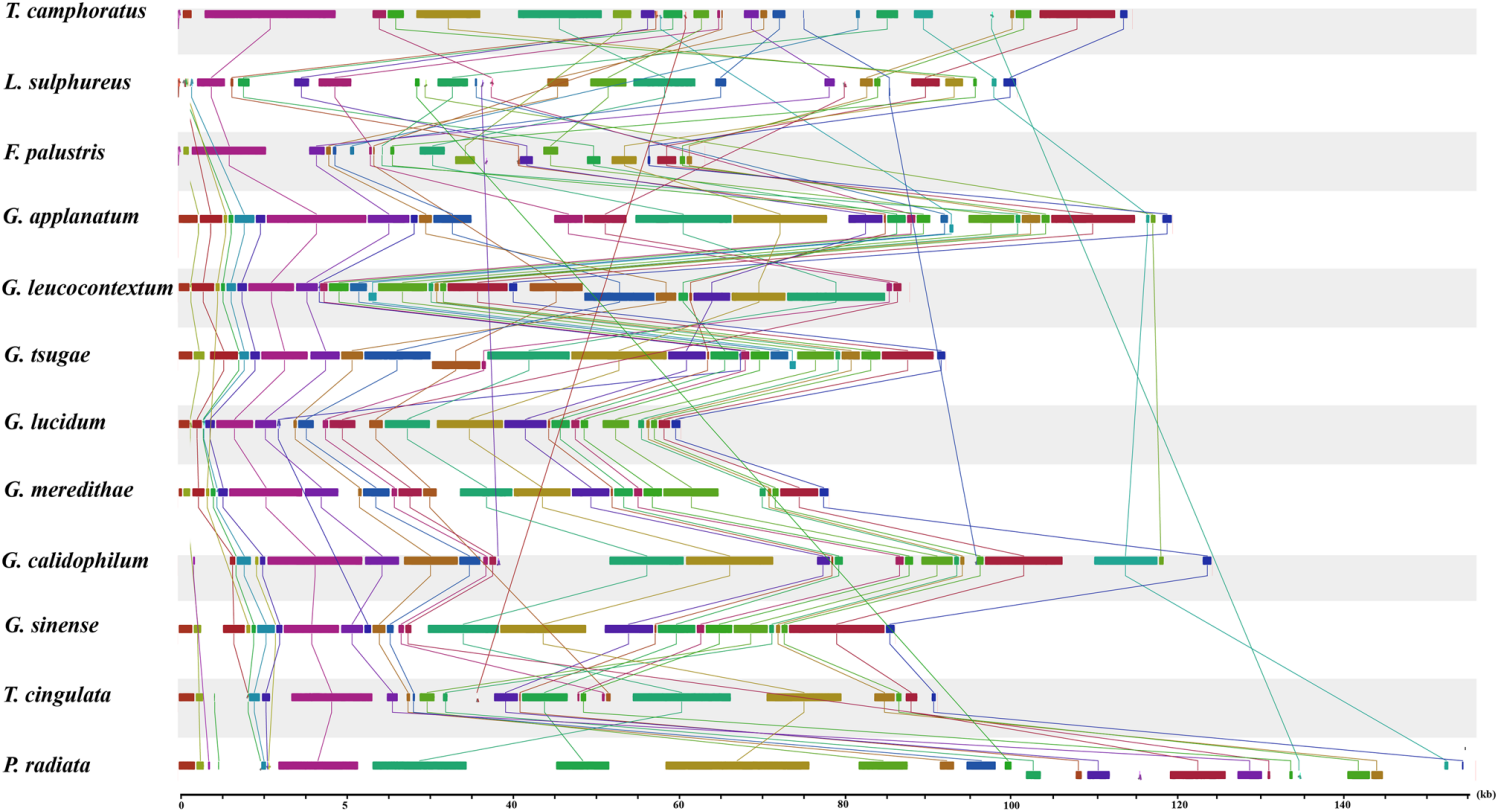
Co-linearity analysis of 12 *Polyporales* mitogenomes. Homologous regions were connected with straight lines of the same color. The sizes and relative positions of the homologous regions varied among the mitogenomes.

Genomic collinearity analysis showed that the mitogenome of *T. camphoratus* had 28 homologous regions with other *Polyporales* mitogenomes (Fig. 5). The relative positions of these homologous regions were highly variable between *Polyporales* species, suggesting that frequent gene rearrangements had occurred during the mitogenome evolution of *Polyporales*. In addition, we found that the gene arrangement was highly conserved in the genus *Ganoderma* and highly variable at family or order levels. Gene arrangement analysis involving all core PCGs, rRNA genes and tRNAs indicated that *T. camphoratus* had a unique gene order, which showed its unique evolutionary characteristics in *Polyporales* species.

### Dynamics of introns in *cox1* and *cob* genes of *Polyporales*

**Figure 6:**
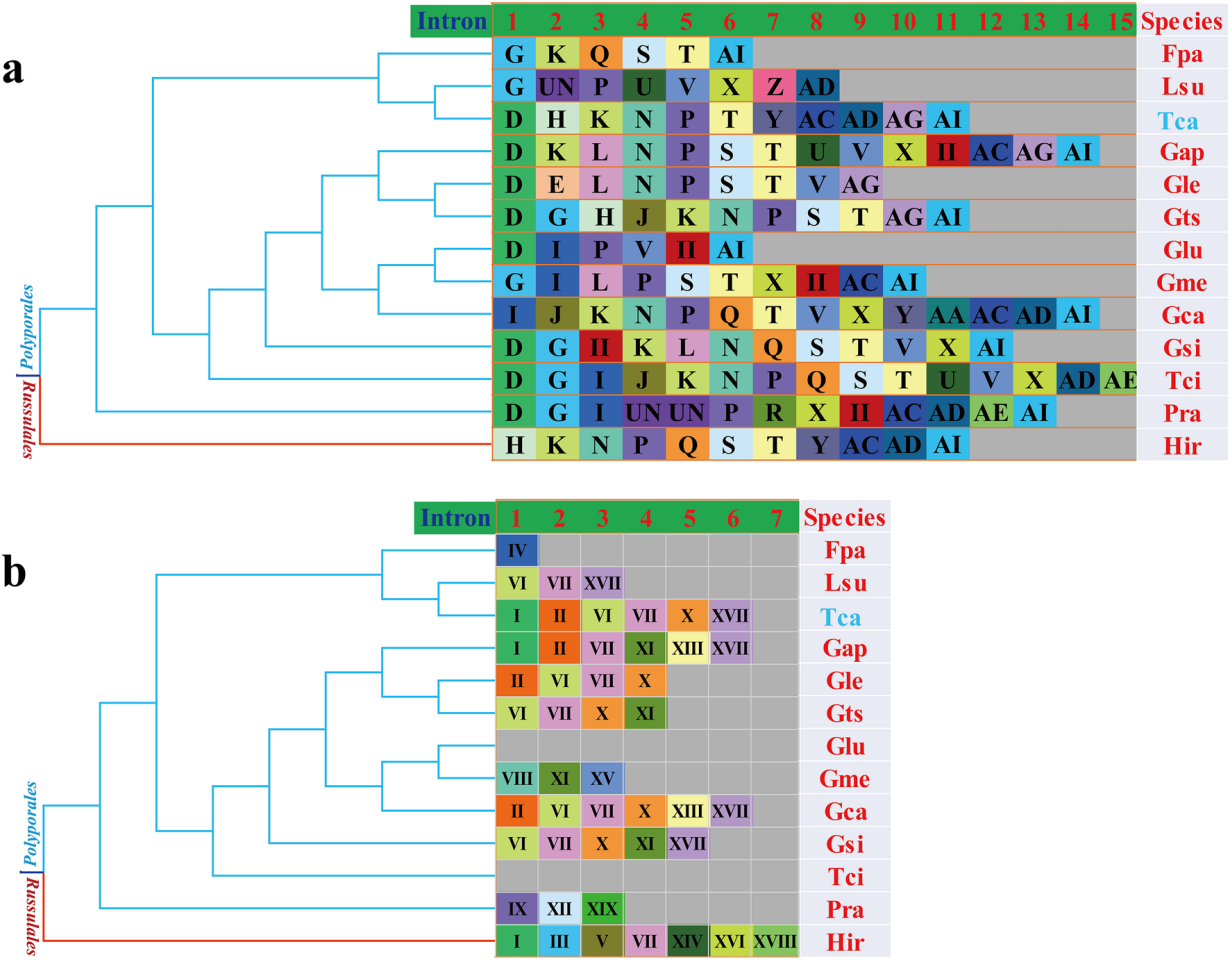
Pcl information of *cox1* gene (**a**) and *cob* gene (**b**) of the 12 *Polyporales* species. The same Pcl (orthologous intron) is represented by the same letter or the same roman numeral. The phylogenetic positions of 12 *Polyporales* species were established using the Bayesian inference (BI) method and Maximum Likelihood (ML) method based on 15 concatenated mitochondrial core proteins and 2 rRNA genes. UN indicates that the intron is different from the known introns^44^ in insertion site and sequence similarity. The II in the figure above shows that the intron belongs to the group II intron. Species ID are shown in Supplementary Table S8.

The number and size of introns were highly variable in mitogenomes of *Polyporales*, which promoted the size and organization dynamic changes of *Polyporales*. A total of 271 introns were detected in 12 published mitogenomes of *Polyporales*, with individual species containing 6–36 introns. These introns distributed in several host genes, including *cox1*, *cob*, *rnl*, *cox2*, and *nad1*. The *cox1* gene contained 130 introns, accounting for 47.97% of the total number of introns, followed by *cob* gene, which contained 48 introns, accounting for 17.71% of the total number of introns. According to previous studies^43^, each intron was considered as a mobile genetic element. Group I introns in fungi could be classified into different position classes (Pcls) according to their insertion site in the coding region of *cox1* or *cob* gene. The same Pcls were considered to be orthologous, and had high nucleotide similarities. Different Pcls had lower sequence similarities, and with non-homologous homing endonucleases. In the present study, we classified group I introns in *cox1* genes of the 12 *Polyporales* species into 26 Pcls according to methods described by Férandon et al.^43^. As shown in Fig. 6, the same letters denoted the same Pcls. *T. cingulata* contained the most Pcls (15) in the *cox1* gene, followed by *G. calidophilum* and *G. applanatum*. *G. lucidum* contained the least Pcls, suggesting that intron loss had occurred in the evolution of *G. lucidum*. Pcls P was the most common intronic classes in the *cox1* genes of *Polyporales*, which presented in 10 of the 12 *Polyporales* species. Pcls T and D were also widely distributed in *cox1* genes of *Polyporales* species, which existed in 9 and 8 *Polyporales* species, respectively. In addition, some rare introns were found in the *cox1* gene of *Polyporales*, e.g. Pcl Z, which was observed only in the *cox1* gene of *L. sulphureus*. However, this Pcls has been observed in the mitogenome of *Agaricus bisporus* from *Agaricales*^43^, suggesting potential horizontal gene transfer events between the two species. Pcl R was observed only in *P. radiata* from *Polyporales*, but was observed in *cox1* gene of *Rhizophydium* sp. 136^43^. In addition, Pcl Y was also considered to be a rare intron class in *Polyporales*, which only existed in *T. camphoratus* and *G. calidophilum*. The Pcl Y was also observed in distant species *Chlorokybus atmophyticus*. In addition, three unknown Pcls were found in the mitogenome of *Polyporales* with no significant nucleotide similarities to reported Pcls.

The introns of *cob* genes in *Polyporales* were classified into 18 Pcls according to the insertion sites in the coding region of *cob* gene (Fig. 6). *T. camphoratus*, *G. applanatum* and *G. calidophilum* contained the most Pcls in *cob* gene. The *cob* gene of *T. cingulata* and *G. lucidum* was intronless. Pcls VI and VII were considered to be the common Pcls in *Polyporales*, which existed in at least 6 of the 12 *Polyporales* species detected. While Pcls IV, VIII, IX, XII, XV, and XIX only existed in one of 12 species, which were considered to be rare introns in the *cob* gene. However, these rare Pcls showed highly nucleotide similarities with distant species, suggesting frequent gain or loss of *cob* introns in *Polyporales*.

### Evolution rate of core genes and phylogenetic analyses

**Figure 7:**
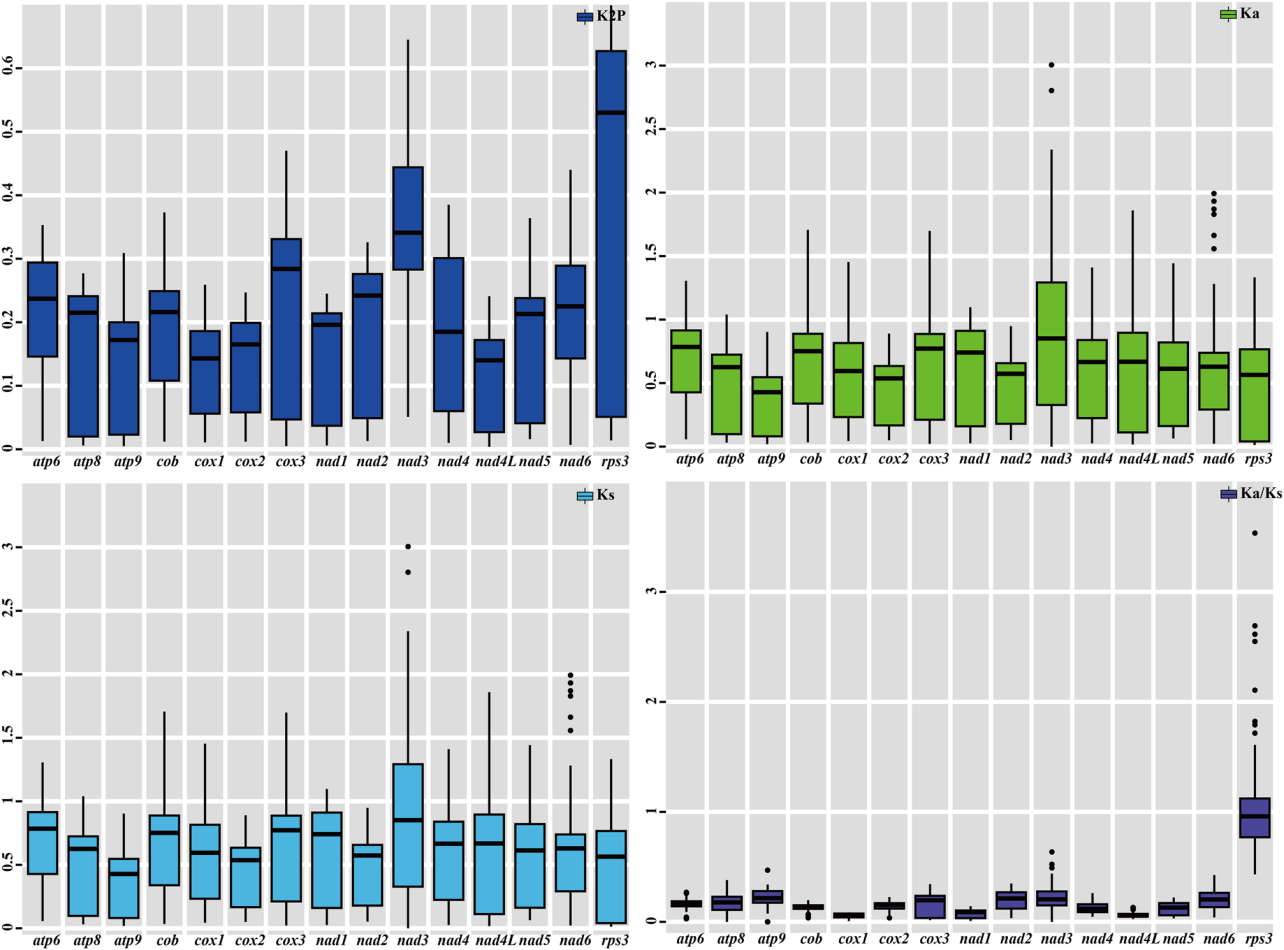
Genetic analysis of 15 protein coding genes conserved in 12 *Polyporales* mitogenomes. *K2P* the Kimura-2-parameter distance, *Ka* the mean number of nonsynonymous substitutions per nonsynonymous site, *Ks* the mean number of synonymous substitutions per synonymous site.

Of the 15 core PCGs detected, the *rps3* gene had the highest mean K2P genetic distance among the 12 *Polyporales* species, followed by *nad3* (Fig. 7). The pairwise K2P genetic distance of *rps3* and *nad3* gene between *Polyporales* species varied largely. The mean genetic distance of *nad4L* gene between the 12 *Polyporales* species was the smallest among the 15 core PCGs detected, indicating that this gene was highly conserved across the mitogenomes. The *nad3* gene had the highest nonsynonymous substitution rate (Ka) of the 15 core PCGs detected, while *atp9* had the lowest rate. The highest synonymous substitutions rate (Ks) was observed in the *nad3* gene, while *atp9* exhibited the lowest Ka value of the 15 PCGs detected. The Ka/Ks values for most core PCGs were less than 1, indicating that these genes were subject to purifying selection. However, the Ka/Ks value of *rps3* gene was observed more than 1 between some species, such as between *T. camphoratus* and *L. sulphureus*, between *T. camphoratus* and *F. palustris*, as well as between *G. sinense* and *G. applanatum*, indicating that *rps3* gene was under positive selection pressure in some *Polyporales* species.

**Figure 8:**
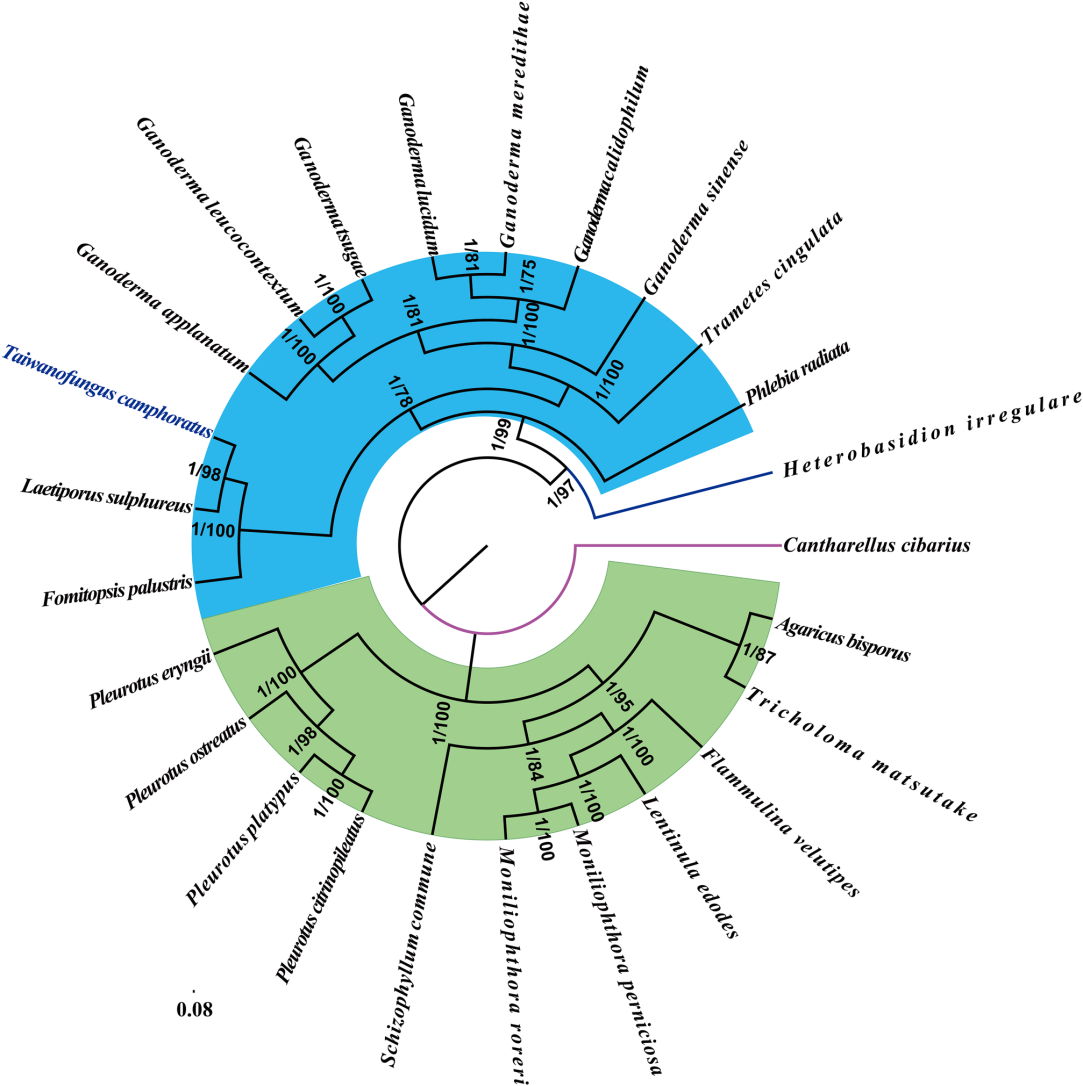
Molecular phylogeny of 25 *Agaricomycetes* species based on Bayesian inference (BI) and Maximum Likelihood (ML) analyses of 15 protein coding genes and two rRNA genes. Support values are bayesian posterior probabilities (before slash) and bootstrap values (after slash). Species and NCBI accession numbers for genomes used in the phylogenetic analysis are provided in Supplementary Table S8.

Phylogenetic analyses based on the combined mitochondrial gene set (15 core PCGs + 2 rRNAs), using bayesian inference (BI) and maximum likelihood (ML) methods with the GTR+I+G model of nucleotide substitution, yielded identical and well-supported tree topologies (Fig. 8). All major clades of the trees were found with good support (BPP = 1.00 and BS ≥ 75). Based on the phylogenetic analyses, the 25 *Agaricomycetes* species could be divided into four major clades, corresponding to the orders *Polyporales*, *Agaricales, Russulales, and Cantharellales* (Table S8). The 12 *Polyporales* species could be divided into three groups; one group was composed of one species in the *Phlebia* genus; the second group was composed of one species in the *Trametes* genus and seven species in the *Ganoderma* genus; the third group was recovered as (*F. palustris* + (*T. camphoratus* + *L. sulphureus*)). *T. camphoratus* was identified as a sister species to *L. sulphureus*. Phylogenetic studies showed the evolutionary status of the *T. camphoratus* in the *Agaricomycetes* class. The results indicated that the combined mitochondrial gene set was suitable as reliable molecular marker for analysis of the phylogenetic relationships among *Agaricomycetes* species.

## Discussion

As the ‘second genome’ of eukaryotes, mitochondrial genome plays an important role in aging, death, disease occurrence and stress resistance of eukaryotes^54–56^. Mitochondrial genome organization, core protein coding genes, repetitive sequences, gene arrangement, the number and structure of tRNAs, and open reading frames provided rich genetic information for understanding the genitics and evolution of eukaryotes^57–59^. Mitochondrial genomes have been extensively studied in animals. However, as an important group of eukaryotes, the mitochondrial genomes of fungi have been less studied, especially in *Agaricomycetes*^60^. As the largest mushroom-forming fungal group, less available *Agaricomycetes* mitochondrial genomes have limited our understanding of the origin, evolution, and phylogenetic relationships of mushroom-forming fungi. The mitochondrial genome of fungi was thought to have a moderate mutation rate, intermediating to that of plants (highest mutation rate) and animals (lowest mutation rate)^61, 62^. It was reported that the mitogenome size, gene arrangement, and repetitive sequences of fungi varied greatly between and within species^39, 47, 48, 63^. The largest known *Agaricomycetes* mitochondrial genome was *Rhizoctonia solani*^64^, which was 235.85 kb long and contained 127 PCGs, 2 rRNA genes and 26 tRNA genes. In *Polyporales* order, the mitogenome size varied greatly, ranging from 60,635 to 156,348 bp. Accordingly, the non-intronic ORFs ranged from 15 to 79; intron numbers ranged from 6 to 36, and the number of tRNA genes ranged from 25 to 29. Variations in the number and size of these genes contributed to huge variations in the size of the mitochondrial genomes in *Polyporales*. Previous studies showed that the size variation of mitogenome was closely associated with intron number variation^43, 44^, which was consistent with this study. In the present study, the *T. camphoratus* genome size, open reading frames, GC content, GC skew and AT skew were not consistent with other *Polyporales* species, indicating the unique evolutionary characteristics of *T. camphoratus* mitogenome.

In the present study, we found that most introns in *Polyporales* belonged to the group I, which was different from plants^61^. A total of 271 introns were found in the 12 *Polyporales* species, most of which were distributed in the protein coding genes *cox1* and *cob*. The dynamic changes of these introns contributed to variations of mitochondrial genome size and organization. Introns were considered to be a mobile genetic element^65^, while homologous introns had the same insertion site in the coding region of PCGs^43^. The same Pcls had high sequence similarities, while different Pcls showed low similarities in nucleotide sequences. In the present study, the classes and number of Pcls were inconsistent in *Polyporales*. Some Pcls were widely distributed in *Polyporales* species, suggesting that these Pcls may be obtained from the common ancestor. However, some Pcls, e.g. Pcls Z and R, were observed only in one or two *Polyporales* species, which were also found in distant species such as *A. bisporus* and *C. atmophyticus*^43^, suggesting the possibility of horizontal gene transfer events. In general, the number and classes of introns were highly dynamic changes in the *Polyporales* species, which proved that numerous intron loss or gain events occurred in the evolution of *Polyporales*. The result was consistent with previous studies that the fungal group I introns appears to result from numerous losses and gains of the mobile genetic elements^40, 43, 66, 67^.

The arrangement of mitochondrial genes can also serve as an important reference for revealing phylogenetic relationships among species^68–70^. In this study, we found that the arrangement of mitochondrial genes was highly variable in *Polyporales*. Gene orders between *Ganoderma* species were found highly conserved, while large-scale gene rearrangements were observed at family levels. Gene order analysis revealed that large-scale gene rearrangements occurred in *T. camphoratus* mitogenome compared with other *Polyporales*, involving protein-coding genes, tRNA genes, and rRNA genes, resulting in unique gene arrangement in *T. camphoratus* mitogenome. Previous studies have shown that the accumulation of repetitive sequences in the mitochondrial genome of fungi contributed to gene recombination of the fungal mitogenome, which in turn led to the rearrangement of mitochondrial genes^61^. In this study, high proportion of repeats (9.44%) were observed in the mitogenome of *T. camphoratus*. It is reasonably speculated that the accumulation of repetitive sequences in the mitogenome of *T. camphoratus* led to gene rearrangement in *T. camphoratus*. The effect of the repeat sequences accumulation in *T. camphoratus* mitogenome on the evolution and variation of *T. camphoratus* mitogenome needs to be further revealed.

Mitogenomes are thought to be derived from the common ancestral alpha-proteobacterium though endosymbiosis^71, 72^. During evolution, most mitochondrial genes have been transferred into the nuclear genome, and this phenomenon was considered with multiple advantages^73, 74^. However, fungal mitogenomes also retained a number of genes for energy metabolism and transcriptional regulation, including *atp6*, *atp8*, *atp9*, *cob*, *cox1*, *cox2*, *cox3*, *nad1*, *nad2*, *nad3*, *nad4*, *nad4L*, *nad5*, *nad6*, and *rps3*, which we call the core PCGs. We found that the core PCGs of *Polyporales* varied greatly in base composition and gene length. The effect of these variations on fungal adaptation to environment and energy metabolism remains unknown. In addition, a series of ORFs were found in the *T. camphoratus* mitogenome, which show low sequence similarities to known proteins in public database, suggesting that there are many unknown protein-coding genes in the *T. camphoratus* mitogenome that need to be revealed, which will facilitate understanding of the origin, evolution and function of fungal mitogenomes. Genetic distance analysis showed that *nad4L* gene was highly conserved among *Polyporales* species, while *rps3* gene was highly variable in *Polyporales*. We found that most of the core PCGs in *Polyporales* were subjected to purifying selection. However, *rps3* gene was under strong positive selection pressure between some species, which may be to better adapt to their lifestyles or surrounding environment^49^.

Because of the limited and confusing macroscopic and microscopic morphological characteristics of macrofungi, it is difficult to precise classify fungal species and subspecies, which limits the development and utilization of fungi. The introduction of molecular markers promotes the taxonomy of fungi. Mitochondrial genomes have been widely used in phylogenetics, evolutionary and population genetics because of rapid evolution rates and many available molecular markers^75, 76^. Mitochondrial *cox1* gene and rRNA genes have been widely used in phylogenetic studies of animals^77, 78^. However, there are few reports on the application of mitochondrial genome in fungal taxonomy or phylogenetics. In the present study, we obtained a high-support phylogenetic tree based on the combined mitochondrial gene set, which divided the 25 *Agaricomycetes* species into four major clades that is consistent with previous studies^1, 20, 63^. *T. camphoratus* was found to be a sister species of *L. sulphureus* and closely related to *F. palustris*^37^*.* The results showed that mitochondrial genes were suitable as molecular markers for phylogenetic analysis of *Agaricomycetes*. More fungal mitochondrial genomes need to be revealed in the future to facilitate the study of fungal taxonomy, phylogenetics and population genetics.

## Materials and methods

### Assembly and annotation of *T. camphoratus* mitogenome

The whole genome sequencing reads of *T. camphoratus* used for mitogenome assembly were downloaded from NCBI SRA database under the accession number (SRR1258102)^7^. De novo assembly of the *T. camphoratus* mitogenome was performed as implemented by SPAdes 3.9^79^ with a kmer size of 17, using the downloaded data. We used MITObim V1.9^80^ to fill gaps among contigs. The obtained complete mitogenome of *T. camphoratus* was initially annotated combining the results of mfannot tool (https://megasun.bch.umontreal.ca/cgi-bin/dev_mfa/mfannotInterface.pl)^81^ and MITOS^82^, both based on the genetic code 4. At this step, protein coding genes (PCGs), tRNA genes and rRNA genes are preliminarily annotated. PCGs were also predicted or modified using the NCBI Open Reading Frame Finder (https://www.ncbi.nlm.nih.gov/orffinder/) and annotated via BLASTP searches against the NCBI non-redundant protein sequence database^83^. PCGs which have no significant similarity to previously characterized proteins were annotated as hypothetical proteins. Intron–exon borders of PCGs were verified using exonerate v2.2^84^. tRNAgenes were also predicted using the tRNAscan-SE 1.3.1 program^85^.

### Sequence analysis

DNASTAR Lasergene v7.1 (https://www.dnastar.com/) was used to analyze the base composition of the *T. camphoratus* mitogenome. Strand asymmetry of the mitogenome was assessed according to the following formulas: AT skew = [A − T]/[A + T], and GC skew = [G − C]/[G + C]^86^. We used the DnaSP v6.10.01^87^ software to calculate the synonymous substitution rate (Ks) and the nonsynonymous substitution rate (Ka) for all core PCGs in the *T. camphoratus* mitogenome, as well as in the previously published *Polyporales* mitogenomes.

The genetic distances between each pair of the 14 core PCGs (*atp6, atp8, atp9, cox1, cox2, cox3, nad1, nad2, nad3, nad4, nad4L, nad5, nad6,* and *cob*) and the ribosomal protein S3 (*rps3*) gene were calculated with MEGA v6.06^88^, using the Kimura-2-parameter (K2P) model. Codon usage were analyzed using the Sequence Manipulation Suite^89^, based on genetic code 4. Genome synteny of the *T. camphoratus* mitogenome and its closely related species were analyzed with the Mauve v2.4.0^90^.

### Identification of repetitive elements

BLASTn searches of the whole mitogenome against itself were performed to determine whether there was intra-genomic duplication of large fragments and interspersed repeats in the *T. camphoratus* mitogenome at an E value of < 10^−10^. In addition, tandem repeats (> 10 bp in length) were detected using the online program Tandem Repeats Finder with default parameters^91^. Repeated sequences were also searched by REPuter to identify forward (direct), reverse, complemented, and palindromic (revere complemented) repeats^92^.

### Comparative mitogenomic analysis and intron analysis

The genome sizes, GC content, base composition, and gene numbers were compared among different *Polyporales* species to assess variations and conservation among mitogenomes. Group I introns in *cox1* and *cob* genes of the 12 *Polyporales* species we detected were classified into different position classes (Pcls) according to the method described by Férandon et al.^44^. Each Pcl was constituted by introns inserted at the same position in the coding region of the *cox1* or the *cob* gene. The Pcls of *cox1* gene were named in letter according to the similarity with the described Pcls^44^. Pcls of *cob* gene were named in Latin number according to the insert position in the coding region of the *cob* gene.

### Phylogenetic analysis

In order to investigate the phylogenetic status of *T. camphoratus* among *Agaricomycetes* class, we constructed a phylogenetic tree of 25 *Agaricomycetes* species based on the combined mitochondrial gene set (15 core PCGs + 2 rRNA genes). Single mitochondrial gene was first aligned using MAFFT v7.037^93^. And then we concatenated these alignments to gene set using SequenceMatrix v1.7.8^94^. In order to detect potential phylogenetic conflicts between different genes, we carried out a preliminary partition homogeneity test. Best-fit models of evolution and partitioning schemes for the gene set were determined according to PartitionFinder 2.1.1^95^. MrBayes v3.2.6^96^ was used to construct the phylogenetic tree using Bayesian inference (BI) method based on the combined gene set. Two independent runs with four chains (three heated and one cold) each were conducted simultaneously for 2 × 10^6^ generations. Each run was sampled every 100 generations. We assumed that stationarity had been reached when estimated sample size (ESS) was greater than 100, and the potential scale reduction factor (PSRF) approached 1.0. The first 25% samples were discarded as burn-in, and the remaining trees were used to calculate Bayesian posterior probabilities (BPP) in a 50% majority-rule consensus tree^37^. We also constructed the phylogenetic tree using maximum likelihood (ML) method based on the combined gene set using RAxML v8.0.0^97^.

### Ethical approval

This article does not contain any studies with human participants performed by any of the authors.

## Supplementary information

Supplementary Tables.

## Data Availability

The *T. camphoratus* mitochondrial genome sequences were submitted to GenBank under accession number MH745717.
